# STMP-UNet: Structure-Aware Target Modeling with Morphology Priors for Low-False-Alarm Detection of Small UAV Targets

**DOI:** 10.3390/s26206630

**Published:** 2026-10-20

**Authors:** Wuqiang Xu, Weihao Shen, Jianlin Zhang, Rongrong Xue, Yang Liu, Haorui Zuo, Haotong Ma

**Affiliations:** 1 State Key Laboratory of Optical Field Manipulation Science and Technology, Institute of Optics and Electronics, Chinese Academy of Sciences, Chengdu 610209, China; xuwuqiang25@mails.ucas.ac.cn (W.X.); shenweihao241@mails.ucas.ac.cn (W.S.); jlin@ioe.ac.cn (J.Z.); xuerongrong@ioe.ac.cn (R.X.); ly5890@outlook.com (Y.L.); 2 University of Chinese Academy of Sciences, Beijing 100049, China; 3 Key Laboratory of Optical Engineering, Chinese Academy of Sciences, Chengdu 610209, China

**Keywords:** single-photon LiDAR, small UAV detection, structure-aware target alignment, morphology prior, false-alarm suppression

## Abstract

**Highlights:**

**What are the main findings?**

- This study proposed STMP-UNet for the low-false-alarm detection of small UAV targets under simulated single-photon observations.
- The STA module suppresses ineffective shallow-response propagation, while the MPM supports the spatial continuity of coarse target-region responses.

**What are the implications of the main findings?**

- Candidate-level FROC analysis shows higher Pd than Pure U-Net at FPPI budgets of 0.05, 0.10, and 0.20.
- Compared with Pure U-Net, STMP-UNet improved Object F1 from 0.7115 to 0.7531 and P_d_ from 0.6635 to 0.7074, while reducing FPPI from 0.1907 to 0.1619.

**Abstract:**

Single-photon LiDAR possesses weak echo sensing capability, but random backgrounds can easily cause target response loss and fragmented false alarms. In particular, sparse return photons, background photons, and dark counts may generate stochastic responses, making weak target preservation and false-alarm suppression difficult under single-photon observations. This paper proposes STMP-UNet, which uses a structure-aware target alignment module to suppress ineffective shallow response propagation through decoder semantic constraints, and combines a morphology prior module to support the spatial continuity of coarse target-region responses. The former selectively transfers target-related shallow information under high-level semantic guidance, while the latter introduces multi-scale structural constraints to alleviate spatial fragmentation of sparse target responses. Experiments are conducted on simulated single-photon data constructed from Anti-UAV infrared sequences. A photon-counting statistical simulation is used to reproduce sparse photon responses, and an infrared-derived proximity map provides auxiliary spatial structural information. Results show that compared to Pure U-Net, STMP-UNet improves Object F1 from 0.7115 to 0.7531 and Pd from 0.6635 to 0.7074, and reduces FPPI from 0.1907 to 0.1619. Ablation and candidate-level FROC analyses further demonstrate the effectiveness of the proposed structure modeling for preserving weak target responses and improving the detection–false-alarm trade-off.

## 1. Introduction

In recent years, “low, slow, and small” unmanned aerial vehicles (UAVs) have been widely applied in inspections, emergency scenarios, and other applications, with increasing demands for their use in the continuous surveillance of airports, critical infrastructures, and low-altitude environments [1]. Due to their small size, weak long-range responses, and complex backgrounds, these UAVs are prone to missed detections and false alarms. Recent studies have improved weak small-target detection through infrared small-target segmentation [2], UAV representation enhancement [3], and attention-based, U-shaped, shape-aware, and related infrared small-target modeling strategies [4,5,6,7]. Single-photon LiDAR provides strong weak-signal sensing capability [8,9], but low signal-to-noise ratio conditions, sparse return photons, background photons, and dark counts may induce random triggers and generate false target responses [10,11]. Therefore, achieving both enhanced weak-target detection and false-alarm suppression remains a key challenge for single-photon “low, slow, and small” UAV detection.

Although existing infrared small-target detection methods have achieved considerable progress under conventional imaging conditions, they do not explicitly address the sparse and stochastic response characteristics of single-photon observations. In particular, shallow features may contain randomly triggered background responses, while sparse target photons can lead to incomplete and spatially fragmented target structures. These characteristics make direct feature transmission and conventional pixel-wise prediction less effective for reliable weak-target detection. U-Net [12] and its variants [13] provide a potential solution through encoder–decoder architectures and cross-level feature fusion. In the evaluated positive frames, the annotation boxes occupy 10–1024 pixels^2^; the task therefore emphasizes single-frame detection and center localization rather than UAV-type recognition or trajectory tracking.

To address these challenges, we propose a Structure-aware Target Modeling and Morphology Prior network (STMP-UNet). The structure-aware target alignment (STA) module formulates shallow random response interference as a cross-scale feature selection problem and achieves selective transmission of target-related information through semantic constraints. The morphology prior module (MPM) addresses spatial fragmentation caused by sparse photon responses by enhancing target region continuity through multi-scale structural constraints. Due to the lack of publicly available single-photon UAV datasets, we construct a dual-channel simulated single-photon UAV dataset based on Anti-UAV infrared sequences [14]. Photon-counting statistical simulation is adopted to reproduce the sparsity of weak returns, while an infrared-derived proximity map provides spatial structural priors. Experimental results demonstrate that STMP-UNet achieves an improved overall balance between target detection and false-alarm suppression compared with Pure U-Net.

**The main contributions of this work are summarized as follows:**

1. **A dual-channel simulated single-photon UAV detection framework is developed by combining photon-event responses with an IR-derived proximity map to provide complementary spatial information.**
2. **STA converts conventional skip connections into decoder-semantic-guided selective feature transmission, with the aim of improving the preservation of weak target-related responses.**
3. **MPM introduces multi-scale morphology priors to support the spatial continuity of coarse target-region responses, enabling a more favorable detection–false-alarm trade-off.**

## 2. Methodology

The proposed methodology consists of dual-channel simulated single-photon observation construction and the STMP-UNet detection framework, including STA, MPM, and joint supervision.

### 2.1. Single-Photon Imaging Principle and Observation Simulation

Single-photon imaging systems are capable of detecting extremely weak optical signals by recording individual photon-arrival events [15]. In a typical SPAD-based system, detectors operate in Geiger mode, where an incident photon can trigger an avalanche that is subsequently quenched and recorded as a detection event. Under weak-return conditions, however, only a small number of signal photons may be detected from the target, while background photons and detector dark counts can generate additional random events. Consequently, target-related responses may become sparse and spatially discontinuous, while stochastic background detections can further obscure weak target structures. These characteristics make reliable weak-target discrimination particularly challenging and motivate the use of computational feature modeling.

Although individual photon events are highly stochastic, target-related responses still exhibit spatial and contextual correlations that distinguish them from randomly triggered background responses. These correlations can be exploited by computational photon-processing methods [16] and deep neural networks for single-photon image and depth reconstruction [17]. Deep neural networks can therefore provide a suitable computational framework for extracting useful representations from sparse single-photon observations. In this work, this capability is further combined with structure-aware feature transmission and morphology-prior modeling to preserve weak target responses and alleviate spatial fragmentation.

**Figure 1:**
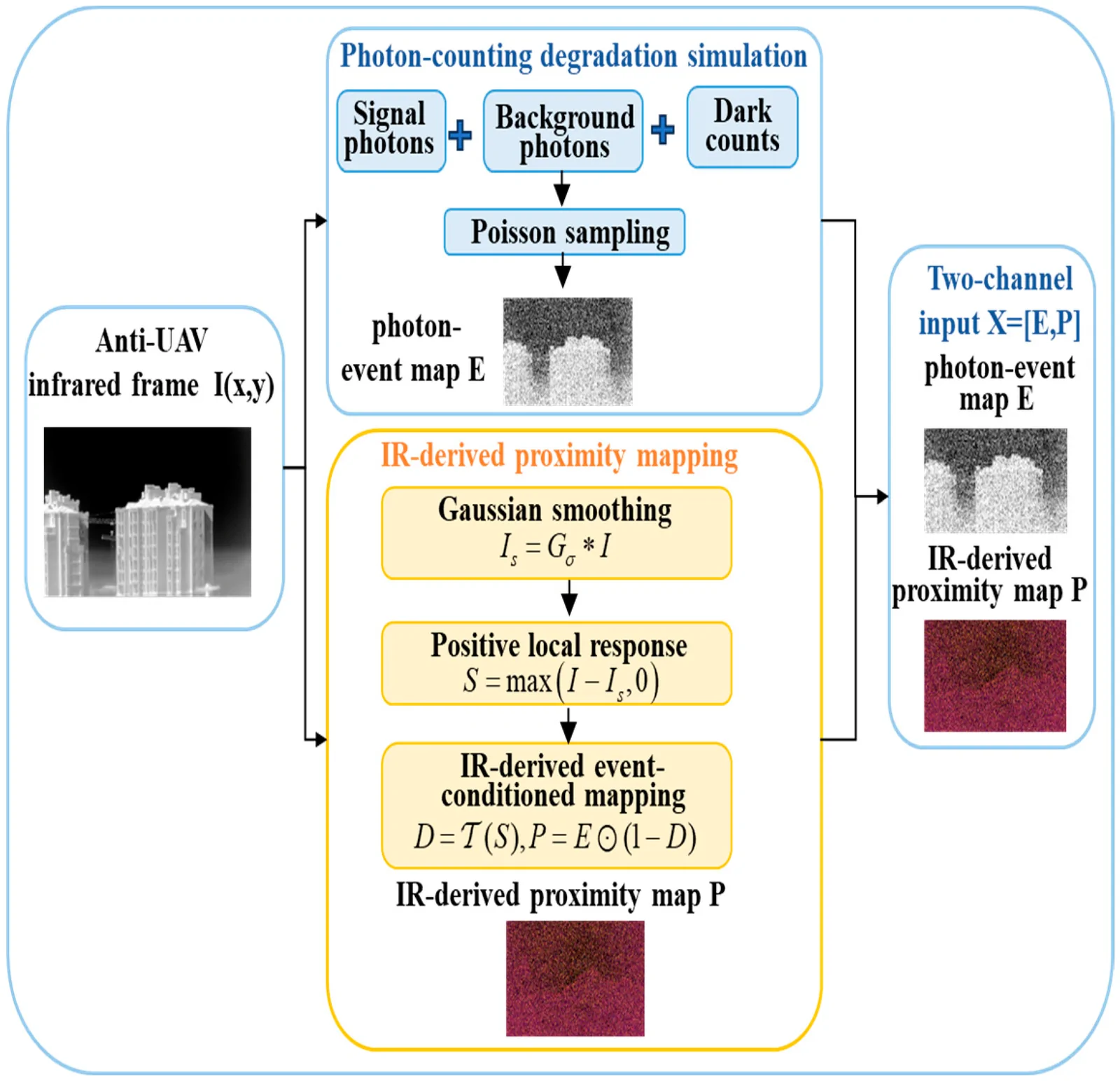
Simulated photon-event generation and IR-derived proximity-map construction.

As shown in Figure 1, this paper constructs a dual-channel simulated single-photon observation based on the normalized infrared frame *I*. Signal photons, spatially non-uniform background photons, and dark counts jointly determine the total photon arrival rate, where the peak signal photon number, average background photon number, and dark count rate are set to 2.0, 0.4, and 0.02, respectively. Photon counting is sampled according to Poisson statistics, and positions where at least one photon count is generated are recorded as binary photon events:

*λ* = 2.0*I* + 0.4(0.5 + *B*) + 0.02,   *K* ∼ Poisson(*λ*),   *E* = **1**[*K* > 0], (1)

where *I* represents the normalized infrared input image, *B* represents the random background field after Gaussian low-pass filtering and normalization, and *λ* represents the expected photon arrival rate at the pixel position. *K* represents the photon count sampled according to the Poisson distribution, and *E* represents the binarized photon event map.

To provide additional spatial structural information for photon events, this paper constructs an auxiliary proximity map *P* based on the original infrared response, which is used together with the photon event map *E* as the network input. This map only represents auxiliary spatial cues derived from the infrared response and does not represent physical time-of-flight (ToF) measurements.

*P* = *E* ⊙ (1 − *D*),   *X* = [*E*,*P*], (2)

where *D* represents the spatial attenuation weight map generated from the infrared response, *P* represents the IR-derived proximity map, and *X* = [*E*,*P*] represents the dual-channel observation input to the network.

For reproducibility, the IR image is divided by 255. The background field is generated by Gaussian smoothing of a uniform random field with a standard deviation of 3 pixels, followed by min–max normalization with ε = 10^−6^. The reference expected-count terms are λs = 2.0I, λb = 0.4(0.5 + B), and λd = 0.02, with total mean λ = λs + λb + λd. These coefficients describe counts per pixel and simulated observation. The dark-count term is additive to the Poisson mean, not a Bernoulli probability or a calibrated rate in Hz. No physical exposure or integration time is assigned.

The IR-derived mapping in Figure 1 is specified as follows. S_0_ = max(I − G_3_*I,0) is min–max-normalized with ε = 10^−6^ and smoothed with G_1_; S is clipped to [0, 1]. The signal-associated proxy is D_0_ = 0.95 − 0.50 S. A signal-associated indicator is sampled at each pixel with probability λs/(λ + ε). For signal-associated pixels, D = clip(D_0_ + η,0,1), where η is zero-mean Gaussian noise with standard deviation 0.02; otherwise, D is sampled uniformly on [0.75, 1]. The final channel is P = E⊙(1 − D). Here, Gσ denotes Gaussian smoothing with standard deviation σ pixels.

P preserves the event locations in E and supplies IR-derived weights, so P-only input is not an independent sensing modality. Input generation uses neither a target mask nor a bounding box. E and P share a source frame and pixel grid in this offline simulation. Reproducing P in a practical system requires access to the auxiliary spatial information; a separate IR source would require synchronization, geometric calibration, and registration with photon measurements. The reported results assume that this auxiliary information is available.

### 2.2. Overall Architecture of STMP-UNet

**Figure 2:**
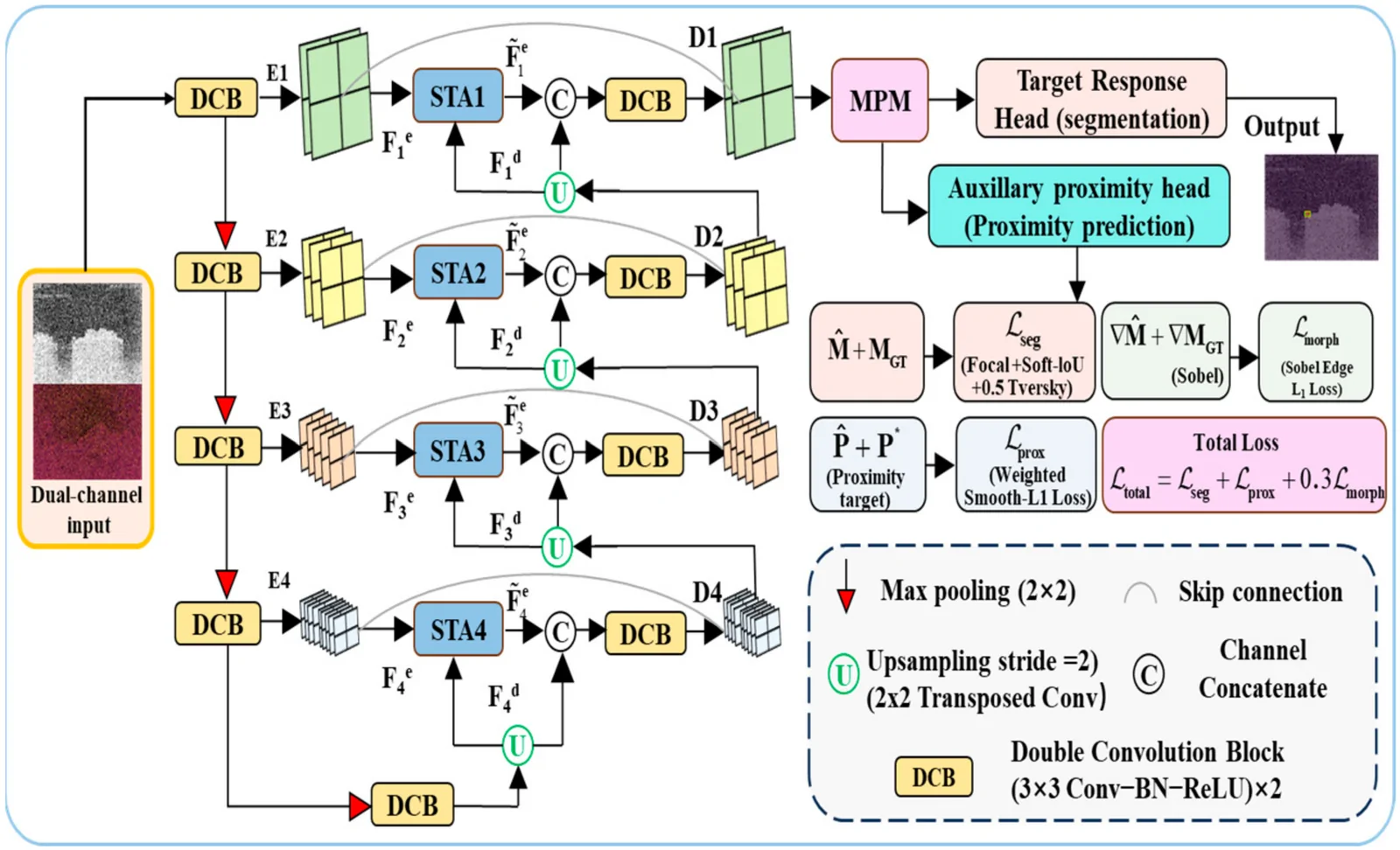
Overall architecture and supervision of STMP-UNet. STA regulates cross-scale skip-feature transmission, while MPM refines the final decoded representation before prediction. The auxiliary target P* shown in the diagram is d* = 0.9 − 0.6 y, distinct from input P. Weighted Smooth-L1 uses foreground/background weights 10/1, while the Sobel term uses 1/0.1 with an external coefficient of 0.3; both are averaged over all pixels.

As shown in Figure 2, STMP-UNet adopts a four-level U-shaped encoder–decoder architecture based on U-Net [12], with the dual-channel observation *X* = [*E*,*P*] as input. STA modules are introduced between the corresponding encoder and decoder stages to regulate the transmission of skip features. After the final decoding stage, MPM further refines the decoded representation before it is fed into the target-response branch and the auxiliary proximity branch.

The encoder progressively extracts multi-scale representations from the dual-channel input, while the decoder restores spatial resolution through hierarchical feature fusion. STA selectively regulates encoder–decoder feature transmission at each corresponding scale, whereas MPM enhances the structural continuity of the final decoded features. The target-response branch produces the final detection map, while the auxiliary proximity branch provides additional spatial supervision during training. The auxiliary-head output is sigmoid-bounded and supervised by a bbox-derived training proxy; it is distinct from input P, and only the target-response head is used for detection.

### 2.3. Structure-Aware Target Alignment (STA)

**Figure 3:**
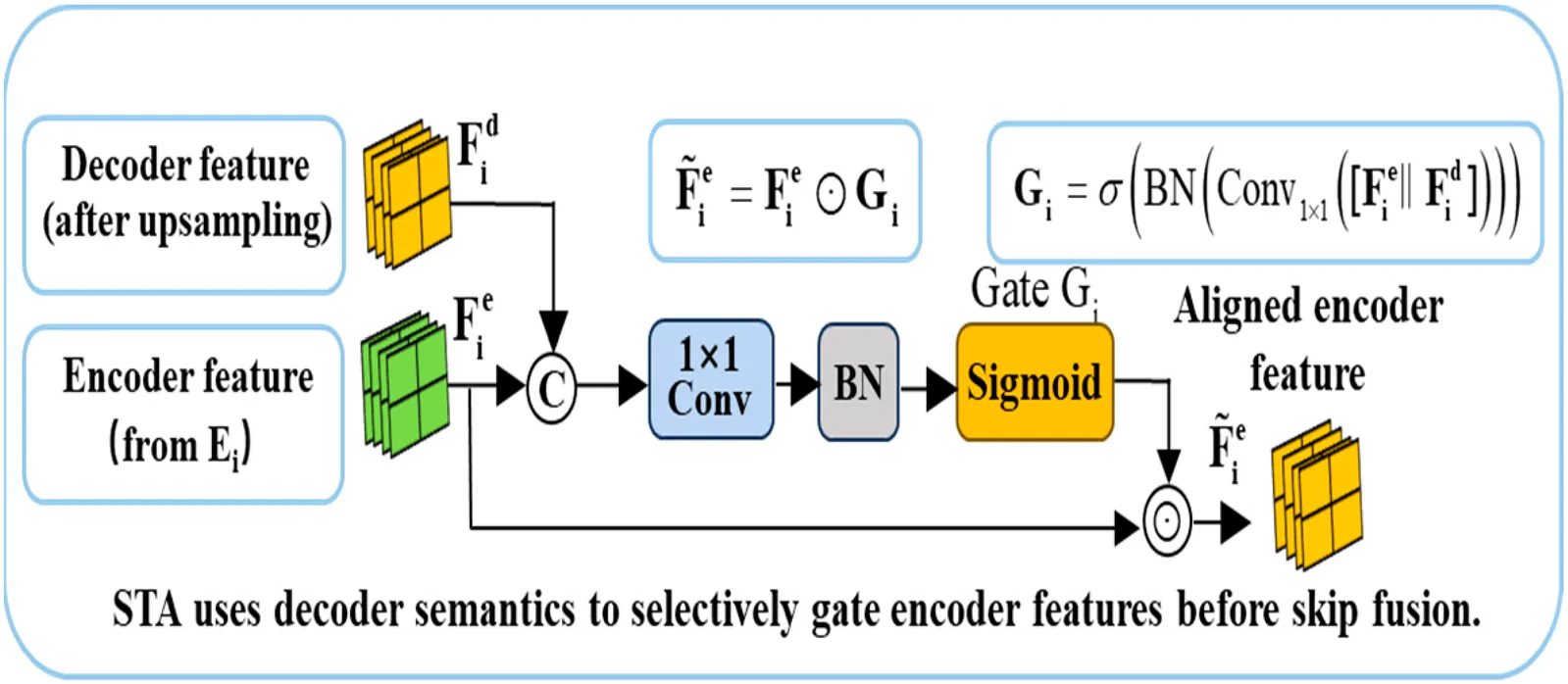
Structure-aware target alignment (STA). Decoder semantic constraints selectively reweight shallow encoder responses before cross-scale feature fusion.

To address the issue that shallow features in single-photon detection often contain random background responses, we introduce the structure-aware target alignment (STA) module, which uses high-level decoder semantics as structural constraints to selectively transfer shallow encoder responses. In this way, conventional indiscriminate skip connections are transformed into semantic-guided transmission of target-related information, as shown in Figure 3. Let the encoder feature at level i be $F_{i}^{e}$, and the corresponding decoder semantic feature at the same scale be $F_{i}^{d}$. STA concatenates the two features and applies a 1 × 1 convolution, batch normalization, and sigmoid activation to generate a gating map that selectively reweights the encoder skip features:

(3) $$G_{i}=\sigma \left(\mathrm{BN}\left[{\mathrm{Conv}}_{1\times 1}\left(F_{i}^{e}\;∥\;F_{i}^{d}\right)\right]\right),\;{\tilde{F}}_{i}^{e}=F_{i}^{e}⊙G_{i},$$

where *G_i_* denotes the gating weight map generated under decoder semantic guidance, ${\tilde{F}}_{i}^{e}$ denotes the selectively enhanced encoder feature, ‖ denotes channel concatenation, and ⊙ denotes element-wise multiplication. This operation enables structural alignment between encoder spatial details and decoder semantic information, allowing target-related shallow responses to be preserved while reducing the propagation of irrelevant random responses.

In this implementation, the STA coefficients vary across both feature channels and spatial locations. Decoder features are bilinearly aligned to the encoder grid when necessary (align_corners = False). Alignment denotes semantic feature selection, rather than geometric registration, temporal alignment, or an independent alignment loss. P influences this operation through encoded features and does not impose a separate explicit geometric constraint. Standard additive Attention Gates [18] instead project the encoder and decoder features separately, sum the projections, apply ReLU, and produce a spatial sigmoid coefficient shared across channels. STA uses concatenation-based fusion and channel-dependent gating within the established attention framework.

### 2.4. Morphology Prior Module

**Figure 4:**
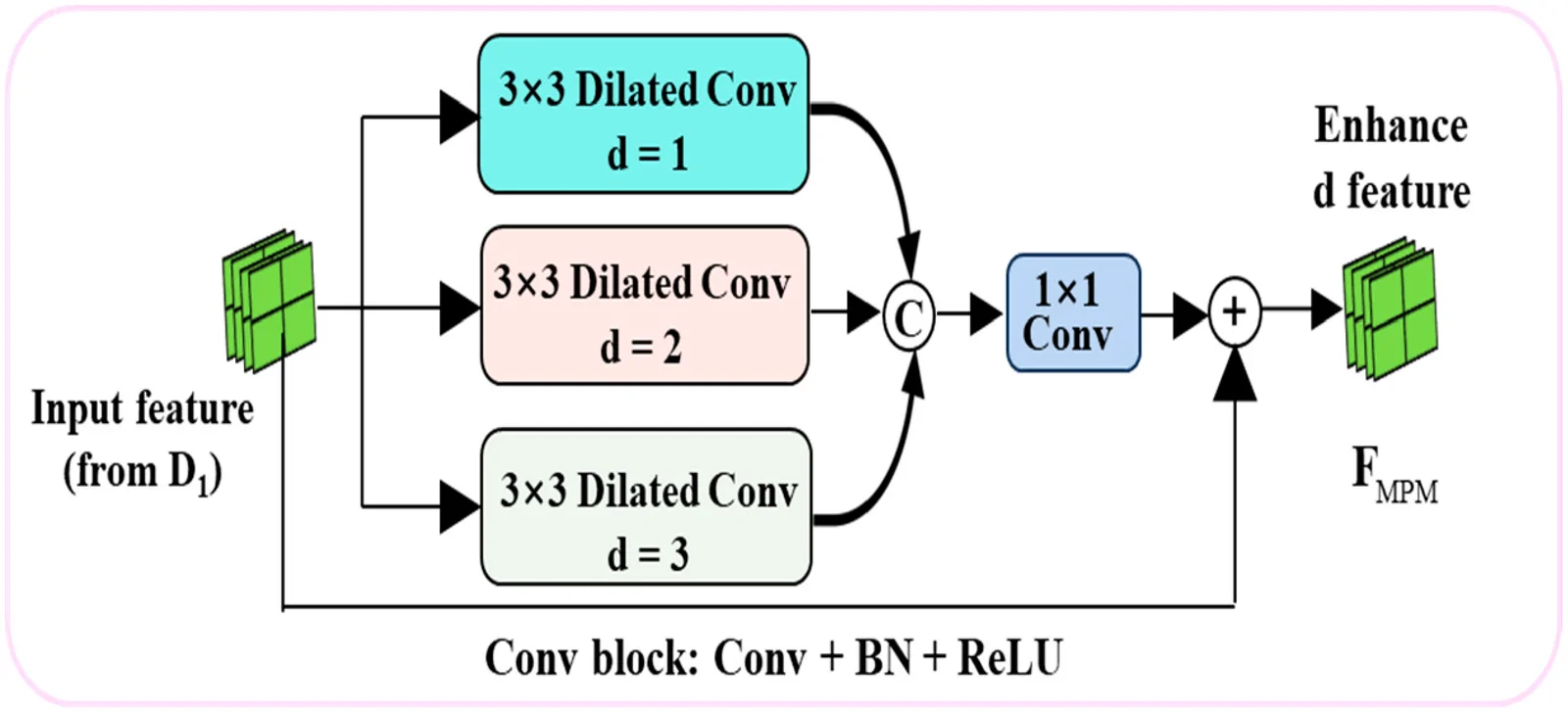
Morphology prior module (MPM). Multi-scale dilated convolutions aggregate local structural information to improve target continuity.

To address the spatial fragmentation issue caused by sparse responses in single-photon detection, we introduce the morphology prior module (MPM) to enhance the structural integrity of target regions. MPM uses multi-scale local structural constraints to supplement spatial continuity information in sparse photon responses, providing implicit morphology priors for weak targets and alleviating fragmented target responses. As shown in Figure 4, MPM is placed after the final decoding stage and consists of three parallel 3 × 3 dilated convolutions with dilation rates d = 1, 2, 3. Their outputs are concatenated, fused through a 1 × 1 convolution, and combined with the input feature through a residual connection to obtain

*F*_MPM_ = *F* + *ϕ*_1×1_[*ϕ*_*d*=1_(*F*) ‖*ϕ*_*d*=2_(*F*) ‖*ϕ*_*d*=3_(*F*)], (4)

where *F* denotes the input feature, *F*_MPM_ denotes the output feature after morphology-prior enhancement, *ϕ_d_* = *ϕ*(●) denotes a 3 × 3 dilated convolution with dilation rate d, *ϕ*_1×1_ = (●) denotes the fusion operation, and ∥ denotes channel concatenation. By aggregating local structures at multiple receptive-field scales, MPM supports the spatial continuity of coarse target-region responses before final prediction.

Each MPM convolutional branch and the 1 × 1 fusion block use batch normalization and ReLU. MPM shares parallel dilated convolution with atrous spatial pyramid pooling (ASPP) [19]. Its present topology uses three local branches and residual fusion, without a separate pointwise or image-pooling branch. Morphology prior refers to an implicit spatial inductive bias; it does not specify a UAV contour model or a mathematical morphology operator.

### 2.5. Loss Function

To jointly optimize target detection, spatial consistency, and structural continuity, STMP-UNet is trained using a composite loss function consisting of focal loss, IoU loss, Tversky loss, proximity loss, and morphology loss. The total loss is defined as

*L*_total_ = *L*_focal_ + *L*_IoU_ + 0.5*L*_Tversky_ + *L*_prox_ + 0.3*L*_morph_, (5)

where *L*_focal_ *L*_IoU_ and *L*_Tversky_ provide direct supervision for target-response prediction. *L*_focal_ focuses the optimization on difficult samples, *L*_IoU_ constrains the overlap between the predicted target region and the ground truth, and *L*_Tversky_ further balances false-positive and false-negative responses. *L*_prox_ provides auxiliary spatial supervision through the proximity branch, while *L*_morph_ penalizes differences in the Sobel gradient magnitude of the predicted target response. Through joint optimization, the network is encouraged to achieve accurate target localization while maintaining spatially coherent responses under sparse single-photon observations.

The component definitions and runtime parameters are given below. Let z denote the target logit, q = sigmoid(z); y ∈ {0, 1} the filled-bounding-box mask; M the batch size; and n the number of pixels per image. Binary cross-entropy is computed from logits as CE = −y log q − (1 − y)log(1 − q), with p_t_ = exp(−CE). Focal loss uses αᵧ = 0.3 for foreground and 0.7 for background, with γ = 2:

(6) $$L_{\mathrm{focal}}=\frac{1}{\mathrm{Mn}}\;\sum_{m,j}\;\alpha_{y}\;(1-p_{t}{)}^{2}\;{\mathrm{CE}}_{m,j}.$$

For image m, let T_m_ = ∑_j_q_mj_y_mj_, F_m_ = ∑_j_q_mj_(1 − y_mj_), and U_m_ = ∑_j_(1 − q_mj_)y_mj_. Soft-IoU and Tversky losses aggregate pixels within each image and then average over images, using ε = 10^−6^:

(7) $$L_{\mathrm{IoU}}=\frac{1}{M}\;\sum_{m}\;[1-\frac{T_{m}+\varepsilon }{\sum_{j}\;q_{m,j}+\sum_{j}\;y_{m,j}-T_{m}+\varepsilon }].$$

(8) $$L_{\mathrm{Tversky}}=\frac{1}{M}\;\sum_{m}\;[1-\frac{T_{m}+\varepsilon }{T_{m}+0.3F_{m}+0.7U_{m}+\varepsilon }].$$

ℒ*_seg_* = ℒ*_focal_* + ℒ*_IoU_* + 0.5ℒ*_Tversky_*. (9)

The auxiliary target is d* = 0.9 − 0.6 y, equal to 0.3 inside the filled annotation rectangle and 0.9 outside it. It is distinct from input P and is not a measured depth or ToF target. With sigmoid-bounded output d^^^, Smooth-L1 uses β = 1 and ℓ(e) = e^2^/2 for |e| < 1; otherwise, ℓ(e) = |e| − 1/2. Foreground/background weights are 10/1:

(10) $$L_{\mathrm{prox}}=\frac{1}{\mathrm{Mn}}\;\sum_{m,j}\;(1+9y_{m,j})l({\hat{d}}_{m,j}-d{*}_{m,j}).$$

The edge term uses K_x_ = [−1,0,1; −2,0,2; −1,0,1] and K_γ_ = [−1,−2,−1; 0,0,0; 1,2,1], with one-pixel zero padding and no kernel normalization by 1/8. The gradient magnitude and weighted absolute error are

(11) $$g(a)=\sqrt{(K_{x}*a{)}^{2}+(K_{y}*a{)}^{2}+10^{-6}}.$$

(12) $$L_{\mathrm{morph}}=\frac{1}{\mathrm{Mn}}\;\sum_{m,j}\;(0.1+0.9y_{m,j})\;|g(q{)}_{m,j}-g(y{)}_{m,j}|.$$

The weighted proxy and edge terms are averaged over all pixels, without division by the weight sum. Their external coefficients in Equation (5) are 1 and 0.3. Because the supervision masks are filled rectangles, the edge term primarily constrains coarse box boundaries and does not establish recovery of true UAV contours.

## 3. Experimental Setup

### 3.1. Dataset

The experiments are conducted on simulated single-photon data constructed from the Anti-UAV infrared dataset using the procedure described in Section 2.1. The dataset contains 45,723 training frames, 3000 validation frames, and 21,155 test frames. The bounding-box annotations provided by Anti-UAV are used to derive rectangular target masks, filter frames, guide training crops, and evaluate detections; they are not involved in generating the simulated single-photon inputs.

The local training/validation/test pools contain 45,723/16,336/21,155 retained frames from 109/48/67 clip directories, drawn from 160/67/91 available directories. The validation subset used for the full-training-set experiments contains 3000 frames. Filtering retains positive frames with a bbox area 10–1024 pixels^2^ and annotated no-target frames, following annotation-frame indices rather than a fixed temporal stride. The complete test set contains 20,002 positive and 1153 negative frames. No-target supervision masks are zero.

### 3.2. Implementation Details

All models were implemented in PyTorch (version 2.1.2, CUDA 12.1) and trained on an NVIDIA GeForce RTX 3090 GPU (NVIDIA Corporation, Santa Clara, CA, USA). All models are implemented in PyTorch and trained on an NVIDIA GeForce RTX 3090 GPU. Training uses 256 × 256 crops; validation and testing retain the native image size, with padding to a multiple of 32. The AdamW optimizer is employed with an initial learning rate of 1 × 10^−4^ and a weight decay of 1 × 10^−5^. For the full-training-set experiments, models are trained for up to 30 epochs with a batch size of 8 and a random seed of 42. The model weights achieving the highest Object F1 on the validation set are selected for subsequent evaluation. Training includes random horizontal flipping and a target-guided crop attempt with probability 0.7 for positive frames. Pure U-Net uses segmentation supervision, whereas STMP-UNet and its structural variants use the complete objective.

### 3.3. Evaluation Metrics

At a fixed decision threshold of 0.9, Object F1, detection probability P_d_, Precision, and false positives per image (FPPI) are adopted as the main evaluation metrics. Considering that the bounding-box annotations in Anti-UAV may cover regions larger than the actual compact UAV responses, object-level matching is performed based on normalized center error (NCE) rather than bounding-box IoU. For a predicted connected component, NCE is defined as

(13) $$\mathrm{NCE}=\frac{{∥c_{p}-c_{g}∥}_{2}}{\sqrt{{w_{g}}^{2}+{h_{g}}^{2}}},$$

where **c***_p_* and **c***_g_* denote the centers of the predicted component and the ground-truth target, respectively. *w_g_* and *h_g_* denote the width and height of the ground-truth bounding box. A predicted component is regarded as a correct detection when NCE ≤ 0.50. P_d_ measures the proportion of successfully detected targets, Precision evaluates the reliability of detected candidates, FPPI measures the average number of false-positive components per image, and Object F1 provides an overall balance between detection sensitivity and false alarms.

Predictions are evaluated as 8-connected components with one-to-one NCE matching. Unmatched components count as false positives, including those on no-target frames.

## 4. Results

This section evaluates STMP-UNet through quantitative comparison, ablation experiments, qualitative analysis, and candidate-level FROC analysis.

### 4.1. Quantitative Comparison

The quantitative results are summarized in Table 1. Compared with Pure U-Net, STMP-UNet improves Object F1 from 0.7115 to 0.7531 and P_d_ from 0.6635 to 0.7074, while Precision increases from 0.7669 to 0.8051 and FPPI decreases from 0.1907 to 0.1619. Under the full-training-set protocol, these results indicate that STMP-UNet improves weak-target detection sensitivity while simultaneously reducing false alarms, achieving a better overall detection–false-alarm trade-off.

### 4.2. Ablation Study

The ablation results in Table 1 demonstrate the respective contributions of STA and MPM. Removing STA decreases P_d_ from 0.7074 to 0.6417, although FPPI is reduced from 0.1619 to 0.0555. This indicates that STA mainly contributes to preserving target-related responses through decoder-semantic-guided selective feature transmission, thereby improving detection sensitivity. In contrast, removing MPM decreases Object F1 from 0.7531 to 0.6976 and P_d_ from 0.7074 to 0.6345, while increasing FPPI from 0.1619 to 0.1746. These results support the contribution of MPM to detection performance; the coarse-region structure diagnostics are reported separately in Section 4.5.5. Overall, the full STMP-UNet provides a more balanced trade-off between target detection and false-alarm suppression. The *w*/*o* STA variant also has slightly higher F1 (0.7548 versus 0.7531), so its relation to Full is an operating-point-dependent sensitivity–false-alarm trade-off.

### 4.3. Qualitative Results

**Figure 5:**
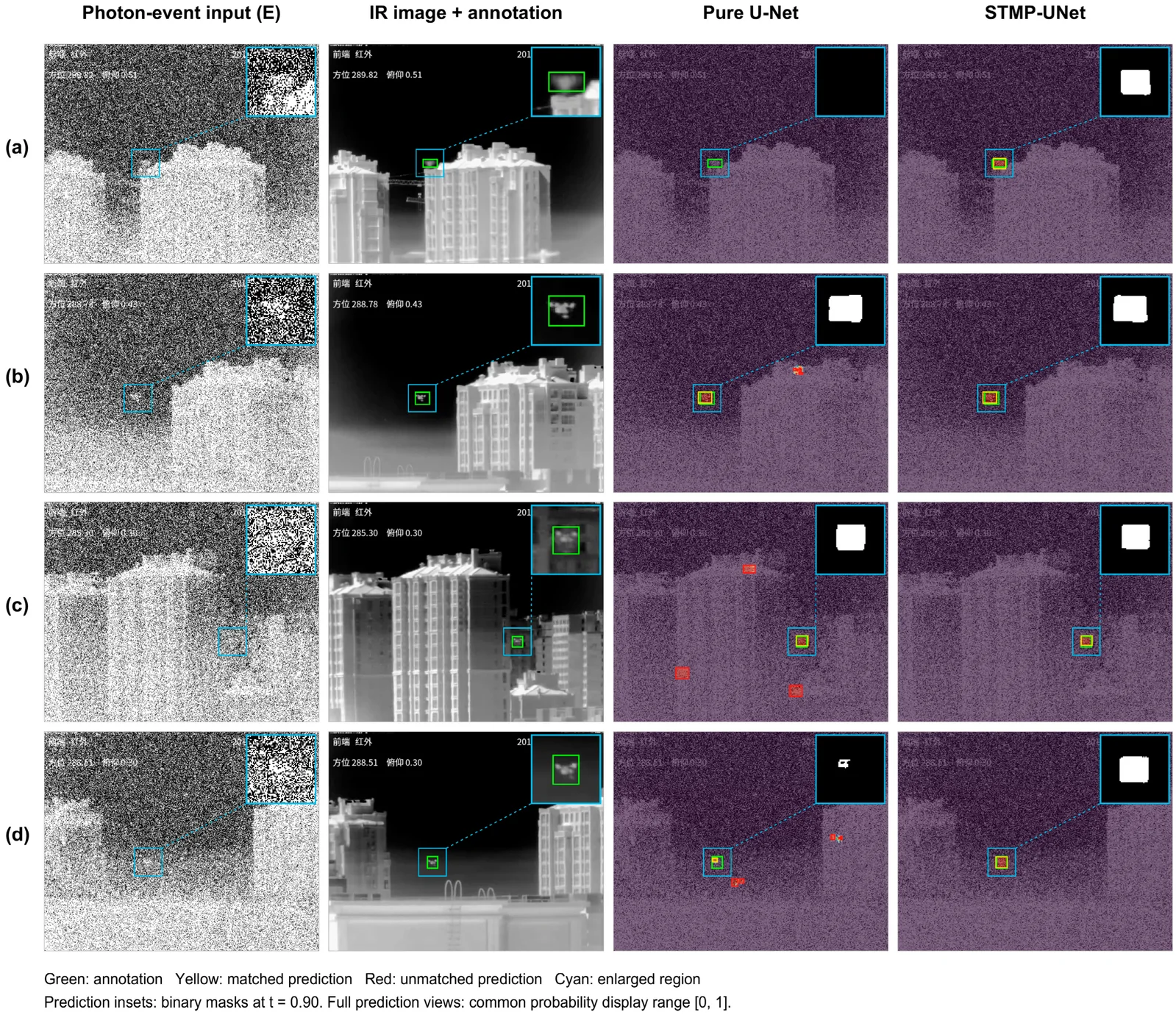
Qualitative comparison of Pure U-Net and STMP-UNet. (**a**) Missed-target recovery; (**b**) suppression of multiple false alarms while preserving target detection; (**c**) suppression of multiple background-induced false alarms in a complex building scene; (**d**) stable target detection with false-alarm suppression under low contrast. Columns show photon-event input, source IR with annotation, Pure U-Net, and STMP-UNet results. Green, yellow, and red boxes indicate targets, correct detections, and false alarms, respectively. Cyan boxes and connectors identify a common 64 × 64-pixel ROI, enlarged by a factor of 2.5. Full-scene probabilities use the same [0, 1] display range; prediction insets show q > 0.90. The acquisition overlays embedded in the source frames contain the Chinese labels “前端”, “红外”, “方位”, and “俯仰”, meaning “front end”, “infrared”, “azimuth”, and “elevation”, respectively. These labels describe acquisition metadata rather than target annotations.

Figure 5 presents qualitative comparisons between Pure U-Net and STMP-UNet in representative challenging scenes. In Figure 5a, Pure U-Net misses the weak target near the building structure, whereas STMP-UNet successfully preserves and detects the target response. In Figure 5b, both models detect the target, but Pure U-Net produces six false-positive components, which are suppressed by STMP-UNet. In Figure 5c, multiple background-induced responses appear in the complex building scene, while STMP-UNet retains the true target and substantially reduces these false detections. Figure 5d further shows that STMP-UNet maintains stable target detection while suppressing discrete false alarms under low-contrast conditions. These qualitative results are consistent with the quantitative results, indicating that STMP-UNet better preserves weak target responses and suppresses background-induced false detections. The insets retain the same coordinates and magnification across columns. These selected examples illustrate detection responses rather than recovery of true UAV contours.

### 4.4. FROC Analysis

**Figure 6:**
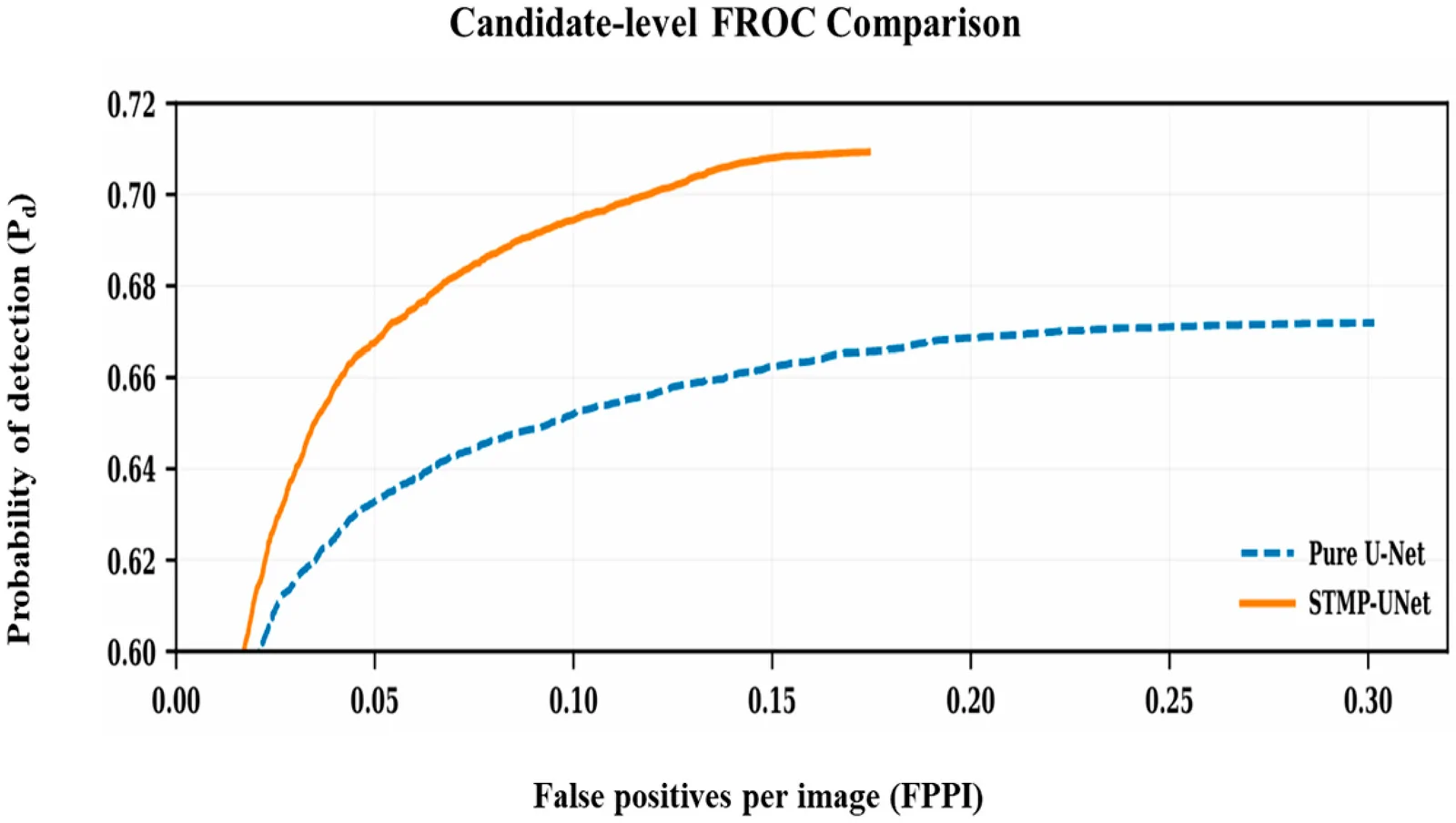
Candidate-level FROC comparison between Pure U-Net and STMP-UNet on the test set. STMP-UNet achieves higher P_d_ at the examined FPPI budgets 0.05, 0.10, and 0.20, indicating a more favorable detection–false-alarm trade-off.

To further evaluate detection performance under low false-alarm conditions, candidate-level free-response receiver operating characteristic (FROC) analysis is performed. A proposal threshold of 0.05 is used to generate candidate detections, and a predicted connected component is regarded as a correct detection when NCE ≤ 0.50. As shown in Figure 6, STMP-UNet achieves higher P_d_ than Pure U-Net at the examined FPPI budgets 0.05, 0.10, and 0.20, indicating that more valid weak-target responses can be retained under strict false-alarm constraints. The FROC results further confirm the improved detection–false-alarm trade-off achieved by STMP-UNet. Candidate confidence is the maximum probability within each connected component. FROC is obtained by sweeping this score while keeping component geometry fixed; tied scores are accepted together. For each FPPI budget, the highest empirical Pd within the budget and the attained FPPI are reported without interpolation.

### 4.5. Performance Analysis

Performance is examined through controlled network comparisons, input ablations, FROC and threshold analyses, robustness tests, structure diagnostics, repeated training, and efficiency measurements. The 3000-frame test subset used in these analyses contains 2836 positive and 164 no-target frames. Training objectives and sample budgets are specified for each analysis; results obtained under different settings are interpreted separately.

#### 4.5.1. Representative Network and Classical Comparisons

A controlled comparison trains freshly initialized STMP-UNet, Pure U-Net, Attention U-Net [18], DNANet [4], and UIU-Net [5] on the same fixed 5000 training and 1000 validation frames, with seed 42 and a maximum budget of 10 epochs. All receive [E,P] and share the segmentation objective, AdamW settings, crops, augmentation, and validation-F1 checkpoint selection. Native segmentation side-output losses are averaged during training, and the primary output is evaluated. The input-layer channel count is adapted to two; STMP-UNet does not use auxiliary proxy or edge supervision in this comparison. The results are summarized in Table 2.

STMP-UNet has the highest reference F1 (0.6855) under this common budget. Compared with the corresponding Pure U-Net control, it has higher Pd and lower FPPI. DNANet has slightly lower FPPI, while Attention U-Net and UIU-Net have higher Pd. These results characterize the specified controlled setting, rather than the fully optimized performance limit of each architecture.

The classical reference applies an elliptical white top-hat transform to uint8 E, followed by Otsu binarization and 8-connected component filtering. A 1000-frame validation grid evaluates radii r ∈ {1, 3, 5} and minimum areas a ∈ {1, 2, 4}; validation F1 selects r = 5 and a = 4, with lower FPPI and a deterministic size rule for ties. No maximum-area filter is used. Table 3 evaluates two parameter settings, r = 3 with a = 1 and validation-selected r = 5 with a = 4, on the same 3000 test frames as the neural models trained on 45,723 frames. Top-hat uses E only; the neural models receive [E,P].

Validation tuning reduces FPPI by approximately 68% relative to r = 3 with a = 1, although Pd also decreases and numerous random-event components remain. The classical result is interpreted as a restricted-input reference, given its different input information.

#### 4.5.2. Contribution of the Auxiliary Input

Pure U-Net and STMP-UNet are trained with E-only, P-only, or E + P inputs, using their respective model-specific objectives, the 45,723-frame training pool, a 3000-frame validation subset, a maximum budget of 30 epochs, and seed 42. All six groups are evaluated on 21,155 test frames (Table 4).

P-only substantially exceeds E-only for both architectures, indicating a major contribution from the IR-derived weights. Pure P-only also exceeds Pure E + P in F1. Full E + P exceeds Full P-only, whereas Full P-only has lower F1 than Pure P-only, with both higher Pd and more false alarms. Pure P-only and Full E + P differ by only approximately 0.16 percentage points in F1, despite different Pd and FPPI. Thus, the auxiliary information and architectural effects should be distinguished, and these single-seed results do not establish statistical significance.

#### 4.5.3. FROC and Threshold Sensitivity

The four-model FROC comparison uses models trained on 45,723 frames and a common proposal threshold of 0.05. Table 5 reports the highest empirical Pd under each budget and the attained FPPI. Full has the highest Pd at the three examined budgets; its gains over *w*/*o* STA are approximately 2.87, 5.15, and 6.65 percentage points. At budget 0.10, however, *w*/*o* STA attains FPPI ≈ 0.0576 while Full attains ≈0.0998. At tighter budgets, 0.001 and 0.005, *w*/*o* STA has higher Pd, so the result does not show a uniform dominance over the full operating range.

For the controlled five-model comparison, a common proposal threshold 0.50 is selected only on validation data from {0.05, 0.30, 0.50, 0.70, 0.90}, maximizing mean Pd across models and budgets {0.05, 0.10, 0.20} at NCE ≤ 0.50, with a lower-threshold tie rule. The same threshold is applied to all five models. This addresses the merging of many DNANet responses into large components at proposal threshold 0.05 without changing model weights or selecting model-specific thresholds on test data.

**Figure 7:**
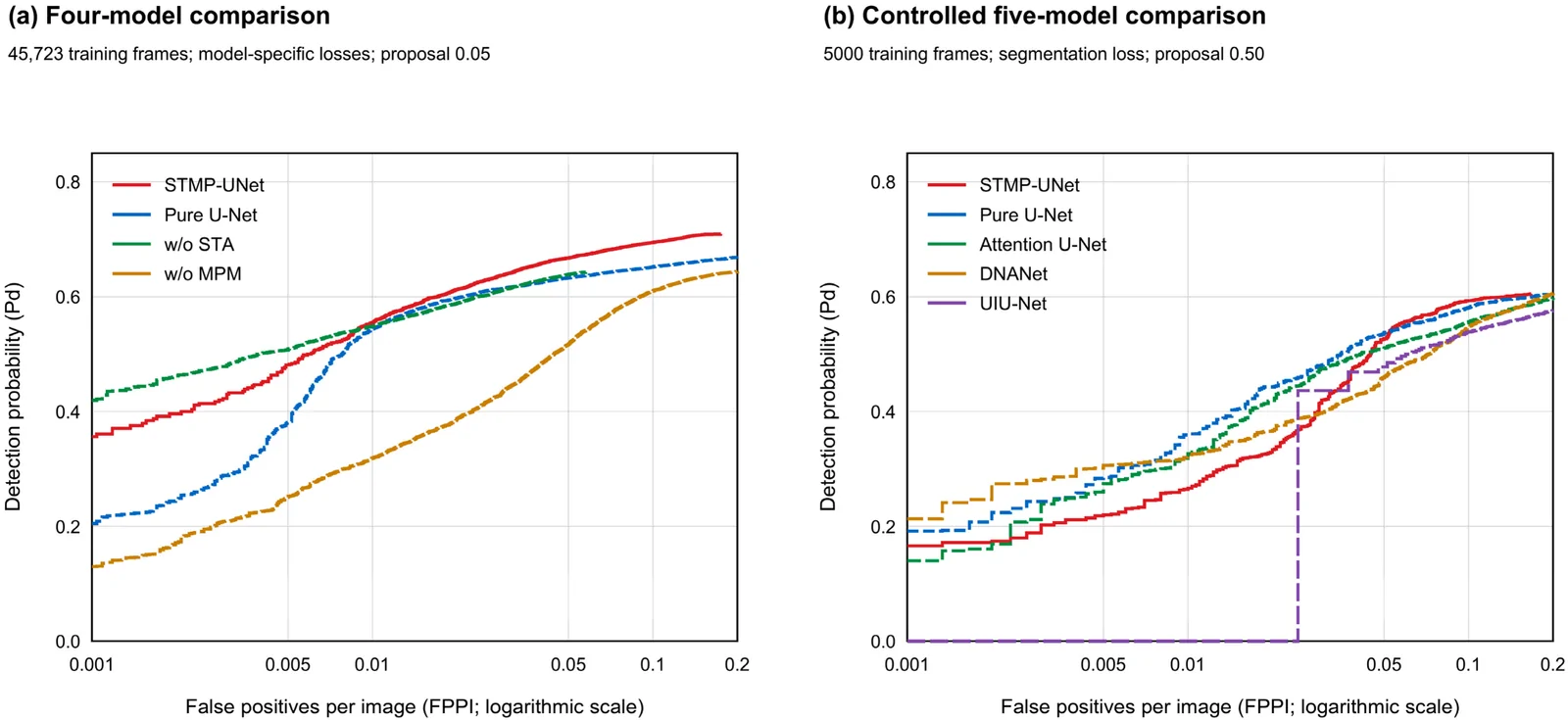
Candidate-level FROC at NCE ≤ 0.50. (**a**) Four models trained on 45,723 frames and evaluated on 21,155 frames with proposal 0.05. (**b**) Controlled five-model comparison on 3000 frames with common proposal 0.50 selected using validation data. The FPPI axis is logarithmic; curves are not extrapolated beyond their attained range. The two protocols are interpreted separately.

In Figure 7b, STMP-UNet attains Pd = 0.5268, 0.5931, and 0.6051 under the three budgets. Pure U-Net is higher at budget 0.05, and DNANet is slightly higher at 0.20.

The threshold-sensitivity results are summarized in Table 6. Across all six threshold/tolerance combinations for the models trained on 45,723 frames, Full remains above Pure U-Net and *w*/*o* MPM in F1 and Pd, with lower FPPI. Its relation to *w*/*o* STA remains a trade-off. Modern-network rankings depend on tolerance: at t = 0.90 and NCE ≤ 0.25, DNANet has F1 = 0.6625 versus 0.6572 for STMP-UNet. Stability within the full-training-set comparison is therefore not generalized to every architecture.

#### 4.5.4. Robustness to Observation Conditions

The Pure U-Net and STMP-UNet models trained on 45,723 frames are tested without retraining on the same 3000 frames. Starting from signal/background/dark-count coefficients 2.0/0.4/0.02, one coefficient is changed at a time. Each condition uses one simulated realization shared by both models. The robustness results are presented in Table 7.

STMP-UNet has higher F1 and Pd and lower FPPI at the reference condition and five perturbations. At signal coefficient 0.5, Pd is 0.2641 versus 0.1164, with fewer false alarms. At background coefficient 1.6, both models have Pd below 1%, and Full no longer retains the F1 or Pd advantage. The tests support relative robustness over the examined conditions and identify failure under stronger backgrounds; they do not establish invariance to arbitrary noise.

#### 4.5.5. Coarse Structure Analysis

On the 2836 positive frames, box coverage is the annotation-rectangle fraction intersecting the q > 0.90 prediction, averaged over all positive frames. A fragmented frame has more than one 8-connected component within this intersection; the fragmentation proportion uses nonempty-overlap frames as its denominator. No NCE matching is used for these diagnostics. Coverage is interpreted alongside object metrics because expanded predictions can increase coverage without improving precision. The coarse annotated-region diagnostics are summarized in Table 8.

Full has greater mean box coverage and less fragmentation than Pure U-Net and *w*/*o* MPM, but *w*/*o* STA has no fragmented overlap in this subset. These findings support coarse region-response modeling rather than superiority in every connectivity measure or recovery of true UAV outlines.

#### 4.5.6. Independent Training Seeds

Full, *w*/*o* STA, and Pure U-Net are independently trained with seeds 42, 43, and 44 on the fixed 5000/1000/3000 subsets for at most 10 epochs. Full and *w*/*o* STA use the complete objective, while Pure U-Net uses segmentation supervision. Mean F1 is 0.6521 ± 0.0184 for Full, 0.6496 ± 0.0194 for *w*/*o* STA, and 0.6625 ± 0.0386 for Pure U-Net (sample standard deviation; *n* = 3). Adding STA reduces both Pd and FPPI in all three seeds, and repeated FROC does not consistently favor Full. These repeats characterize variability under the 5000-frame training protocol and do not establish statistically significant differences between models or uncertainty estimates for the 45,723-frame training protocol.

#### 4.5.7. Network Efficiency

Efficiency is measured on the RTX 3090 with batch size 1 and 512 × 640 dual-channel FP32 input, using 50 warm-up passes and 100 timed forward passes recorded by CUDA events. cuDNN TF32 is enabled; matrix-multiplication TF32 and automatic mixed precision are disabled. Median latency and reciprocal FPS include executed network branches but exclude acquisition, simulation, decoding, and full post-processing. MAC counts cover Conv2d, ConvTranspose2d, and Linear layers and exclude other operators; they are not total-network FLOPs. The coarse annotated-region diagnostics are summarized in Table 9.

STMP-UNet is faster than the three modern baselines in this configuration, but slower than Pure U-Net and its two module-removal variants; DNANet also has fewer parameters. The results describe a performance–cost trade-off, rather than the smallest or fastest architecture or an end-to-end sensor acquisition rate.

## 5. Discussion

Under the 45,723-frame training protocol, the experimental results demonstrate that STA and MPM play complementary roles in STMP-UNet. The STA module mainly contributes to preserving target-related responses through decoder-semantic-guided selective feature transmission. Although removing STA results in a lower FPPI, it also causes a clear decrease in P_d_. This behavior suggests that the no-STA variant produces a more conservative response pattern, reducing false detections at the cost of missing more true targets. Therefore, STA mainly contributes to preserving target-related responses and improving detection sensitivity rather than independently minimizing FPPI.

The MPM addresses a different aspect of the problem by enhancing the spatial continuity of sparse target responses. Removing MPM decreases both Object F1 and P_d_ while increasing FPPI, suggesting that multi-scale structural constraints help maintain more coherent target responses and reduce fragmented detections. Together, STA and MPM enable STMP-UNet to achieve a more favorable balance between weak-target preservation and false-alarm suppression, which is also supported by the candidate-level FROC results. Here, structural continuity concerns responses under rectangle-based supervision; it does not imply recovery of the true UAV contour.

It should be noted that the present experiments are based on simulated single-photon observations derived from Anti-UAV infrared sequences rather than measurements acquired by a real single-photon imaging system. Although the simulation incorporates photon-counting statistics, background responses, and dark-count-like disturbances, it cannot fully reproduce all characteristics of practical single-photon sensors and imaging environments. In addition, the IR-derived proximity map provides auxiliary spatial information and does not represent physical ToF measurements. Therefore, further evaluation using real single-photon measurements is required to assess the generalization of the proposed method under practical sensing conditions. Practical transfer additionally requires validation of the auxiliary-input source and its consistency with photon observations. The reported two-dimensional detection results do not determine sensing distance, ranging accuracy, or deployment performance.

The analyses in Section 4.5 distinguish the contributions of input information, architecture, and operating-point selection. P provides a substantial contribution, modern-network rankings depend on the metric and matching tolerance, and repeated training on 5000 frames does not establish consistent Full superiority. The conclusions therefore apply to the evaluated input and training conditions. The independence of adjacent frames across the training and validation subsets has not been fully verified.

## 6. Conclusions

This paper proposes STMP-UNet for small UAV detection under simulated single-photon observations, aiming to balance weak-target response preservation and false-alarm suppression. The STA module uses decoder-semantic-guided selective feature transmission to preserve target-related shallow responses, while the MPM introduces multi-scale morphology priors to enhance the spatial continuity of sparse target responses. Under the 45,723-frame training protocol, experimental results show that, compared with Pure U-Net, STMP-UNet improves Object F1 from 0.7115 to 0.7531 and P_d_ from 0.6635 to 0.7074, while reducing FPPI from 0.1907 to 0.1619. Under that protocol, ablation experiments, qualitative comparisons, and candidate-level FROC analysis further demonstrate the complementary contributions of STA and MPM and the improved detection–false-alarm trade-off achieved by the proposed method. Future work will evaluate STMP-UNet using real single-photon measurements and further investigate its generalization from simulated observations to practical sensing conditions. The performance analyses in Section 4.5 delineate input, training-budget, and operating-point dependencies without establishing superiority in every metric or statistical significance.

## Figures and Tables

**Table 1:** Quantitative comparison and ablation results. Neural models are evaluated on 21,155 frames at t = 0.90 and NCE ≤ 0.50.

Method	Object F1	Pd	Precision	FPPI
Pure U-Net	0.7115	0.6635	0.7669	0.1907
*w*/*o* STA	0.7548	0.6417	0.9162	0.0555
*w*/*o* MPM	0.6976	0.6345	0.7746	0.1746
**STMP-UNet**	0.7531	0.7074	0.8051	0.1619

**Table 2:** Controlled modern-network comparison on the same 3000 test frames (5000 training/1000 validation frames; at most 10 epochs; seed 42; segmentation-only supervision; t = 0.90; NCE ≤ 0.50).

Method	Object F1	Pd	Precision	FPPI
STMP-UNet	0.6855	0.5987	0.8017	0.1400
Pure U-Net	0.6657	0.5896	0.7645	0.1717
DNANet	0.6667	0.5709	0.8011	0.1340
UIU-Net	0.5096	0.6488	0.4196	0.8483
Attention U-Net	0.6335	0.6125	0.6560	0.3037

**Table 3:** Classical reference and neural models trained on 45,723 frames on matched 3000 test frames (NCE ≤ 0.50). The neural threshold is 0.90; r = 5 and a = 4 are selected on validation data.

Method	Input	Object F1	Pd	FPPI
Top-hat r = 3, a = 1	E	0.000492	0.4743	1822.2430
Top-hat r = 5, a = 4	E	0.000850	0.2623	582.3593
Pure U-Net	E + P	0.7241	0.6752	0.1793
STMP-UNet	E + P	0.7567	0.7130	0.1620

**Table 4:** Input ablations on the 21,155-frame test set (t = 0.90; NCE ≤ 0.50). All input configurations use 45,723 training frames, 3000 validation frames, and a maximum budget of 30 epochs.

Model and Input	Object F1	Pd	Precision	FPPI
Pure E	0.4339	0.5829	0.3456	1.0438
Pure P	0.7516	0.6748	0.8480	0.1144
Pure E + P	0.7115	0.6635	0.7669	0.1907
Full E	0.4492	0.6055	0.3570	1.0310
Full P	0.7318	0.7044	0.7614	0.2087
Full E + P	0.7531	0.7074	0.8051	0.1619

**Table 5:** Four-model candidate FROC on 21,155 frames (proposal 0.05; NCE ≤ 0.50). Brackets report attained FPPI; empirical points are not interpolated.

Model	Pd [FPPI] Budget ≤ 0.05	Pd [FPPI] Budget ≤ 0.10	Pd [FPPI] Budget ≤ 0.20
Pure U-Net	0.6329 [0.0499]	0.6520 [0.0999]	0.6686 [0.1992]
STMP-UNet	0.6675 [0.0498]	0.6943 [0.0998]	0.7093 [0.1743]
*w*/*o* STA	0.6388 [0.0500]	0.6428 [0.0576]	0.6428 [0.0576]
*w*/*o* MPM	0.5167 [0.0499]	0.6101 [0.1000]	0.6437 [0.2000]

**Table 6:** Threshold sensitivity on 21,155 test frames for models trained on 45,723 frames. Pure and Full denote Pure U-Net and STMP-UNet, respectively.

t	NCE	Pure F1	Full F1	Pure Pd	Full Pd	Pure FPPI	Full FPPI
0.85	0.50	0.7116	0.7535	0.6647	0.7078	0.1923	0.1617
0.90	0.50	0.7115	0.7531	0.6635	0.7074	0.1907	0.1619
0.95	0.50	0.7133	0.7539	0.6616	0.7071	0.1829	0.1594
0.85	0.25	0.7033	0.7475	0.6569	0.7022	0.1997	0.1671
0.90	0.25	0.7033	0.7471	0.6559	0.7018	0.1979	0.1672
0.95	0.25	0.7047	0.7483	0.6536	0.7018	0.1904	0.1645

**Table 7:** Fixed-weight robustness on 3000 matched frames (t = 0.90; NCE ≤ 0.50). One simulation coefficient varies at a time.

Condition	Pure F1	Full F1	Pure Pd	Full Pd	Pure FPPI	Full FPPI
Reference 2/0.4/0.02	0.7241	0.7567	0.6752	0.7130	0.1793	0.1620
Signal 0.5	0.1558	0.3426	0.1164	0.2641	0.3570	0.2623
Signal 1.0	0.5664	0.6734	0.5092	0.6100	0.2730	0.1907
Signal 4.0	0.6949	0.7705	0.6738	0.7186	0.2510	0.1387
Background 0.8	0.3801	0.4607	0.3456	0.3646	0.4470	0.2063
Background 1.6	0.0166	0.0059	0.0095	0.0032	0.1303	0.0627
Dark-count term 0.10	0.6835	0.7449	0.6308	0.6946	0.2033	0.1610

**Table 8:** Coarse annotated-region diagnostics on 2836 positive frames (t = 0.90). Fragmentation uses nonempty overlaps; no NCE matching is applied.

Model	Mean Box Coverage	No-Overlap Frames	Fragmented/ Nonempty	Fragmented Proportion
Pure U-Net	54.97%	918	16/1918	0.83%
STMP-UNet	61.37%	814	8/2022	0.40%
*w*/*o* STA	55.72%	995	0/1841	0.00%
*w*/*o* MPM	51.19%	1026	29/1810	1.60%

**Table 9:** Network forward efficiency on RTX 3090 (batch size 1; 512 × 640 dual-channel input). MACs cover Conv/Linear layers; timings exclude acquisition and full pre/post-processing.

Model	Parameters (M)	Conv/Linear MACs (G)	Median latency (ms)	Network FPS
STMP-UNet	7.987	79.063	13.673	73.13
Pure U-Net	7.763	60.262	7.850	127.39
Attention U-Net	34.878	331.936	31.404	31.84
DNANet	4.697	69.598	158.259	6.32
UIU-Net	50.541	271.298	36.082	27.71
*w*/*o* STA	7.812	76.378	12.209	81.91
*w*/*o* MPM	7.956	68.996	10.749	93.03

## Data Availability

The source infrared data used in this study are available from the Anti-UAV dataset [14]. The simulated single-photon observations were generated from the source infrared sequences using the procedure described in this article. Further information is available from the corresponding authors upon reasonable request.
